# Status of the InChI algorithm and InChI trust

**DOI:** 10.1186/1758-2946-4-S1-P13

**Published:** 2012-05-01

**Authors:** Stephen Heller

**Affiliations:** 1 Gaithersburg, MD USA

## 

Progress of the IUPAC InChI/InChIKey project continues to move ahead quote well. Use of the algorithm has increased over the past year to the point that numerous publications use and refer to InChI. The Markush and Polymer/Mixtures IUPAC InChI working groups have completed their initial work and/or are moving ahead at a good pace. Publicity for the project is good and has resulted in considerable increase in the usage of the InChI algorithm. The Trust web site is now available to the public and is updated regularly. Even CAS now allows for an InChI string to be used as input for a SciFinder search. Extensions to the project’s current capabilities are being developed by a number of expert, experienced individuals and groups from various areas of chemistry. This presentation will describe the current technical state of the InChI algorithm and how the InChI Trust is working to assure the continued support and delivery of the InChI algorithm.

